# Supplementary material for: Unlocking the potential of allogeneic Vδ2 T cells for ovarian cancer therapy through CD16 biomarker selection and CAR/IL-15 engineering
Source: Nat Commun. 2023 Nov 8;14:6942. doi: 10.1038/s41467-023-42619-2 (PMC10632431; doi:10.1038/s41467-023-42619-2)
Supplement: Supplementary file 1 — Supplementary Information [file 41467_2023_42619_MOESM1_ESM.pdf]

Supplementary Information for

Unlocking the potential of allogeneic Vδ2 T cells for ovarian cancer therapy through CD16 biomarker selection and CAR/IL-15 engineering

Authors

Derek Lee[1,†], Zachary Spencer Dunn[1,2,†], Wenbin Guo[3,†], Carl J. Rosenthal[1], Natalie E. Penn[1], Yanqi Yu[1], Kuangyi Zhou[1], Zhe Li[1], Feiyang Ma[4], Miao Li[1], Tsun-Ching Song[1], Xinjian Cen[1], Yan-Ruide Li[1], Jin J. Zhou[5], Matteo Pellegrini[3,4,6], Pin Wang[2], and Lili Yang[1,6,7,8*]

Affiliations

[1] Department of Microbiology, Immunology & Molecular Genetics, University of California, Los Angeles, CA, USA.

[2] Mork Family Department of Chemical Engineering and Materials Science, University of Southern California, Los Angeles, CA, USA.

[3] Bioinformatics Interdepartmental Program, University of California, Los Angeles, CA, USA.

[4] Department of Molecular, Cell, and Developmental Biology, University of California, Los Angeles, CA, USA.

[5] Department of Biostatistics, Fielding School of Public Health, University of California, Los Angeles, CA, USA.

[6] Eli and Edythe Broad Center of Regenerative Medicine and Stem Cell Research, University of California, Los Angeles, CA, USA.

[7] Jonsson Comprehensive Cancer Center, David Geffen School of Medicine, University of California, Los Angeles, CA, USA.

[8] Molecular Biology Institute, University of California, Los Angeles, CA, USA.

[†] These authors contributed equally: Derek Lee, Zachary Spencer Dunn, Wenbin Guo

[*]Correspondence: liliyang@ucla.edu; Tel.: (+1-310-825-8609)

The SI includes Supplementary Figures 1-5.

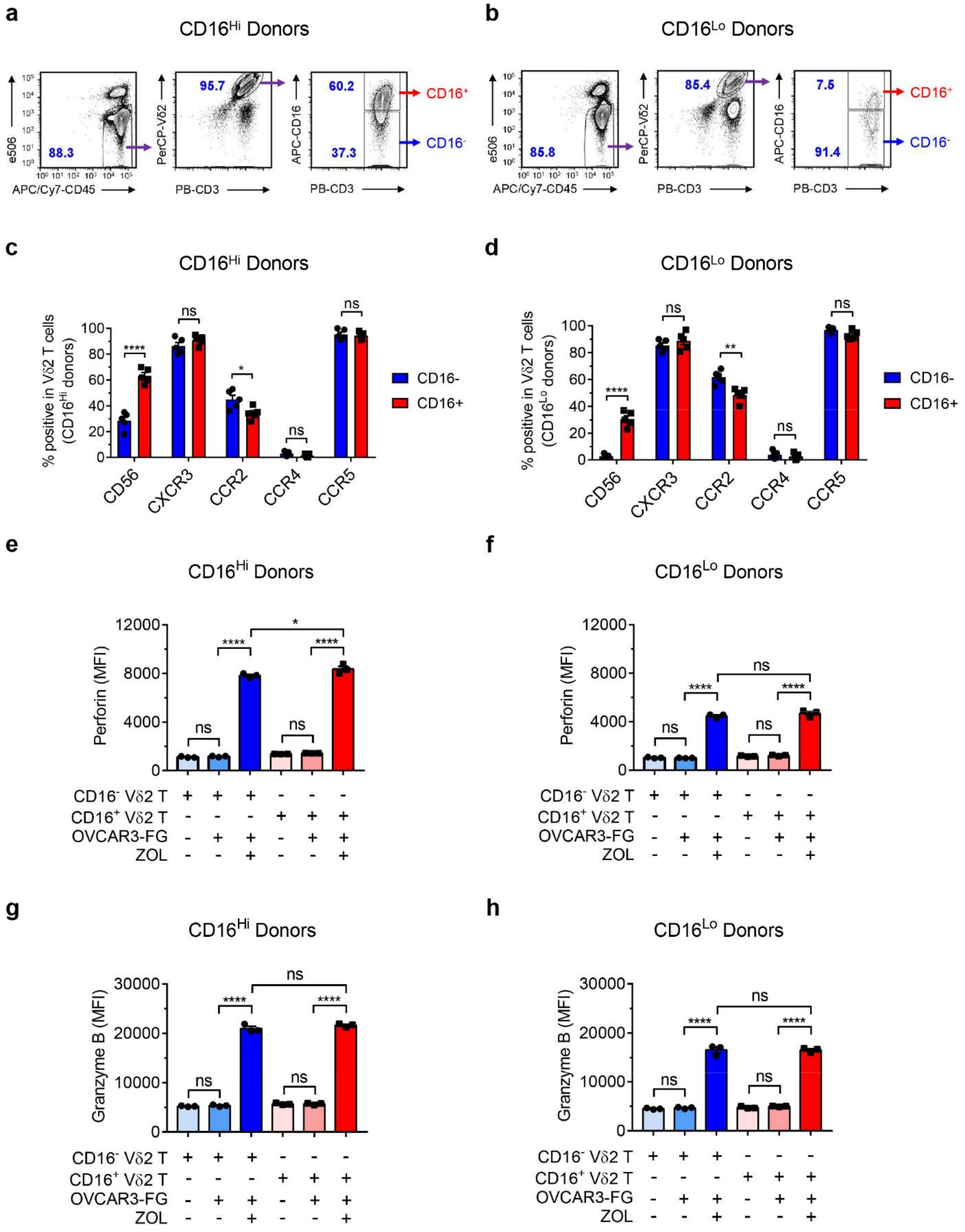

a
CD16Hi Donors
88.3
95.7
60.2
37.3
CD16+
CD16-
e506
APC/Cy7-CD45
PerCP-Vδ2
PB-CD3
APC-CD16
b
CD16Lo Donors
85.8
85.4
7.5
91.4
c
CD16Hi Donors
% positive in Vδ2 T cells (CD16Hi donors)
CD56
CXCR3
CCR2
CCR4
CCR5
CD16-
CD16+
d
CD16Lo Donors
% positive in Vδ2 T cells (CD16Lo donors)
e
CD16Hi Donors
Perforin (MFI)
CD16- Vδ2 T
CD16+ Vδ2 T
OVCAR3-FG
ZOL
f
CD16Lo Donors
g
CD16Hi Donors
Granzyme B (MFI)
h
CD16Lo Donors

Supplementary Fig. 1 Characterization of the CD16$^{+/-}$ Vδ2 T cells generated from the CD16^{Hi} or CD16^{Lo} PBMC donors. Vδ2 T cells were generated from the CD16^{Hi} or CD16^{Lo} donor PBMCs following the experimental design shown in Fig. 1a, then were subject to FACS analysis for **a, b** the gating strategy to select CD16^{+} and CD16^{-} Vδ2 T cells from CD16^{Hi} or CD16^{Lo} donors, **c, d** surface expression markers (n = 5), and **e-h** intracellular perforin and granzyme B production at 24 h after tumor co-culture (E:T ratio = 1:1; n = 3 from 3 different donors). CD16^{+} and CD16^{-} Vδ2 T cells were gated based on cell surface CD16 expression. Representative of >10 (**a, b**), 2 (**c, d**), and 3 (**e-h**) experiments. Data are presented as the mean ± SEM. ns, not significant; *$p < 0.05$; **$p < 0.01$; ****$p < 0.0001$ by Student's *t* test (**c, d**) or by one-way ANOVA (**e-h**). Source data and exact p values are provided as a Source Data file.

a

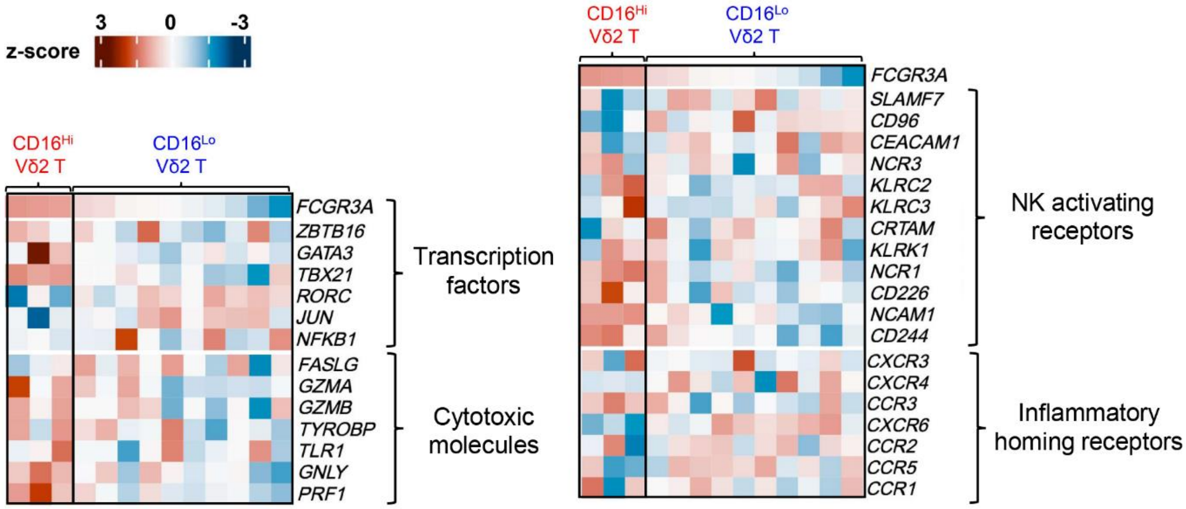

3 0 -3
z-score
CD16Hi Vδ2 T
CD16Lo Vδ2 T
FCGR3A
ZBTB16
GATA3
TBX21
RORC
JUN
NFKB1
Transcription factors
FASLG
GZMA
GZMB
TYROBP
TLR1
GNLY
PRF1
Cytotoxic molecules
CD16Hi Vδ2 T
CD16Lo Vδ2 T
FCGR3A
SLAMF7
CD96
CEACAM1
NCR3
KLRC2
KLRC3
CRTAM
KLRK1
NCR1
CD226
NCAM1
CD244
NK activating receptors
CXCR3
CXCR4
CCR3
CXCR6
CCR2
CCR5
CCR1
Inflammatory homing receptors

b

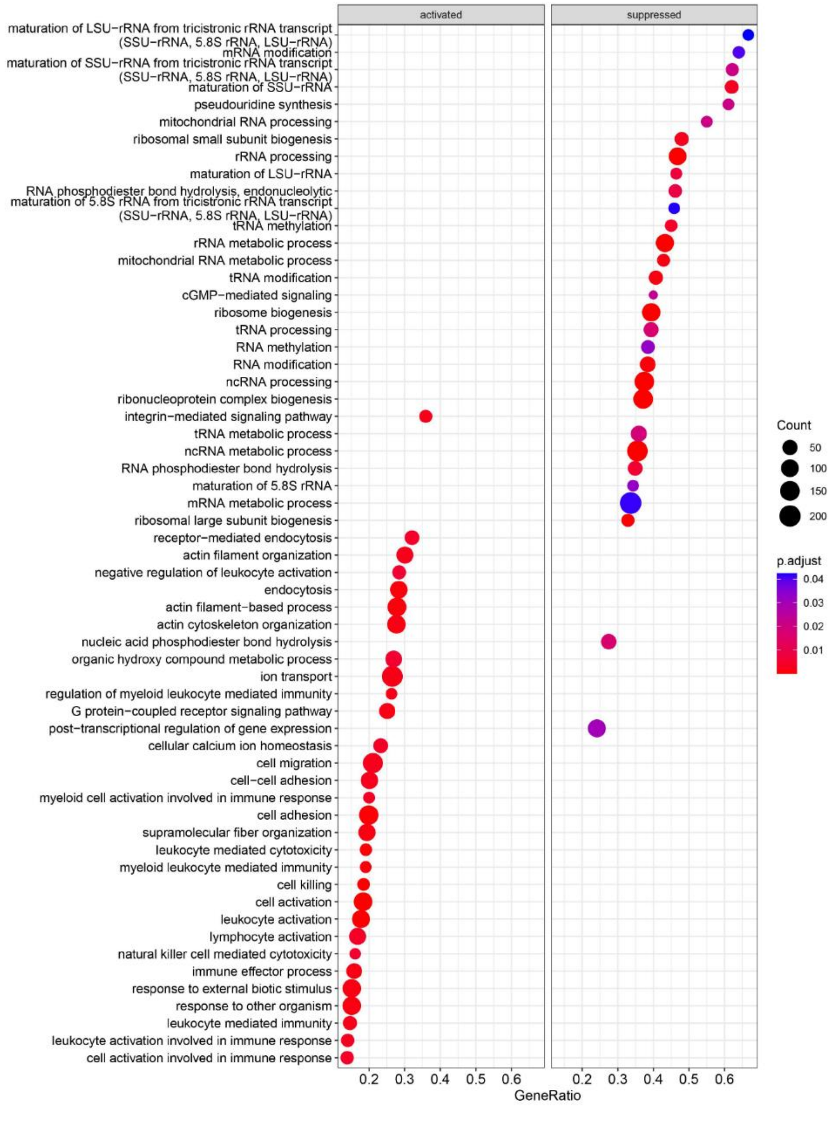

activated
suppressed
maturation of LSU−rRNA from tricistronic rRNA transcript (SSU−rRNA, 5.8S rRNA, LSU−rRNA)
mRNA modification
maturation of SSU−rRNA from tricistronic rRNA transcript (SSU−rRNA, 5.8S rRNA, LSU−rRNA)
maturation of SSU−rRNA
pseudouridine synthesis
mitochondrial RNA processing
ribosomal small subunit biogenesis
rRNA processing
maturation of LSU−rRNA
RNA phosphodiester bond hydrolysis, endonucleolytic
maturation of 5.8S rRNA from tricistronic rRNA transcript (SSU−rRNA, 5.8S rRNA, LSU−rRNA)
tRNA methylation
rRNA metabolic process
mitochondrial RNA metabolic process
tRNA modification
cGMP−mediated signaling
ribosome biogenesis
tRNA processing
RNA methylation
RNA modification
ncRNA processing
ribonucleoprotein complex biogenesis
integrin−mediated signaling pathway
tRNA metabolic process
ncRNA metabolic process
RNA phosphodiester bond hydrolysis
maturation of 5.8S rRNA
mRNA metabolic process
ribosomal large subunit biogenesis
receptor−mediated endocytosis
actin filament organization
negative regulation of leukocyte activation
endocytosis
actin filament−based process
actin cytoskeleton organization
nucleic acid phosphodiester bond hydrolysis
organic hydroxy compound metabolic process
ion transport
regulation of myeloid leukocyte mediated immunity
G protein−coupled receptor signaling pathway
post−transcriptional regulation of gene expression
cellular calcium ion homeostasis
cell migration
cell−cell adhesion
myeloid cell activation involved in immune response
cell adhesion
supramolecular fiber organization
leukocyte mediated cytotoxicity
myeloid leukocyte mediated immunity
cell killing
cell activation
leukocyte activation
lymphocyte activation
natural killer cell mediated cytotoxicity
immune effector process
response to external biotic stimulus
response to other organism
leukocyte mediated immunity
leukocyte activation involved in immune response
cell activation involved in immune response
0.2 0.3 0.4 0.5 0.6
0.2 0.3 0.4 0.5 0.6
GeneRatio
Count
50
100
150
200
p.adjust
0.04
0.03
0.02
0.01

Supplementary Fig. 2 Transcriptome characterization of Vδ2 T cells in relation to CD16 expression. a Heatmaps showing the expression levels of the selected genes in Vδ2 T cells generated from three $CD16^{Hi}$ PBMC donors and ten $CD16^{Lo}$ PBMC donors. **b** Gene set enrichment analysis showing the relationship between CD16 expression and biological process pathways in Vδ2 T cells generated from all 13 PBMC donors (including the three $CD16^{Hi}$ donors and ten $CD16^{Lo}$ donors). The top 30 most significantly activated or suppressed biological process pathways are shown. Representative of 1 experiment.

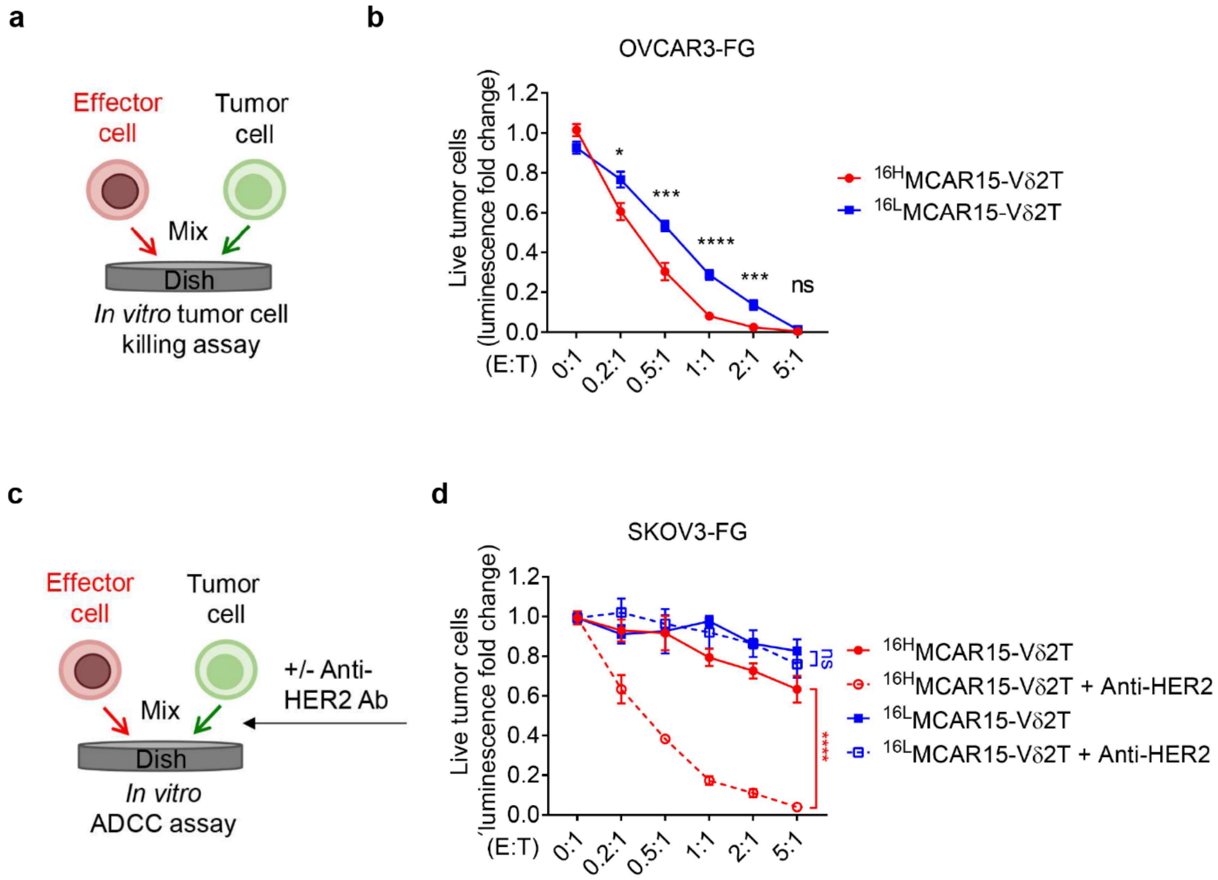

Supplementary Fig. 3 Comparing the *in vitro* antitumor efficacy of CD16^{Hi} and CD16^{Lo} MCAR15-Vδ2T cells. Vδ2 T cells were cultured from the CD16^{Hi} or CD16^{Lo} donor PBMCs and were engineered to express MCAR and IL-15. The resulting cell products are denoted as ^{16H}MCAR15-Vδ2T or ^{16H}MCAR15-Vδ2T cells, respectively. **a, b** *In vitro* tumor cell killing assay. An OVCAR3-FG human ovarian cancer cell line was used. **a** Experimental design. **b** Tumor cell killing data collected as 24 h after co-culture (n = 3; n indicates different donors). **c, d** *In vitro* antibody-dependent cell-mediated cytotoxicity (ADCC) assay. A SKOV3-FG human ovarian cancer cell line was used. **c** Experimental design. **d** Tumor cell killing data collected at 24 h after co-culture (Anti-HER2 Ab concentration = 0.1 μg/mL; n = 3; n indicates different donors). Representative of 3 experiments. Data are presented as the mean ± SEM. ns, not significant; *p < 0.05; ***p < 0.001; ****p < 0.0001 by Student's *t* test (**b, d**). Source data and exact p values are provided as a Source Data file.

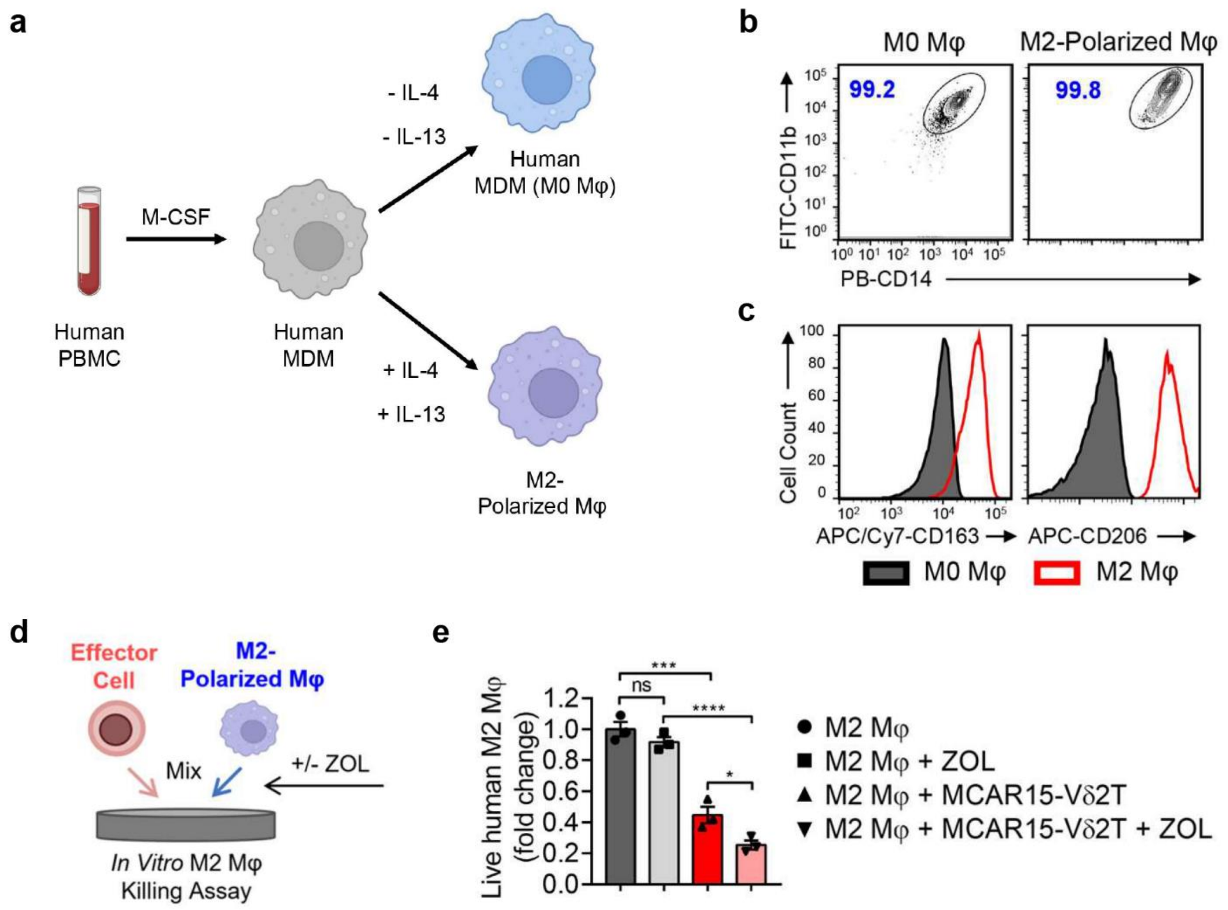

Supplementary Fig. 4 MCAR15-Vδ2T cells can target M2-polarized human macrophages. a Experimental design to generate human monocyte-derived macrophages (MDM), either non-polarized (M0) or M2-polarized. M-CSF, macrophage colony-stimulating factor; Mφ, macrophage. **b** FACS detection of CD11b and CD14 expression on M2-polarized macrophages. **c** FACS detection of M2 macrophage markers (i.e., CD163 and CD206) on M2-polarized macrophages. **d, e** Studying the *in vitro* killing of M2-polarized macrophages by MCAR15-Vδ2T effector cells. **d** Experimental design. **e** Data collected at 24 h after co-culture (E:Mφ ratio = 1:1, n = 3). Data are presented as the mean ± SEM. ns, not significant; *$p < 0.05$; ***$p < 0.001$; ****$p < 0.0001$ by one-way ANOVA (**e**). Source data and exact p values are provided as a Source Data file.

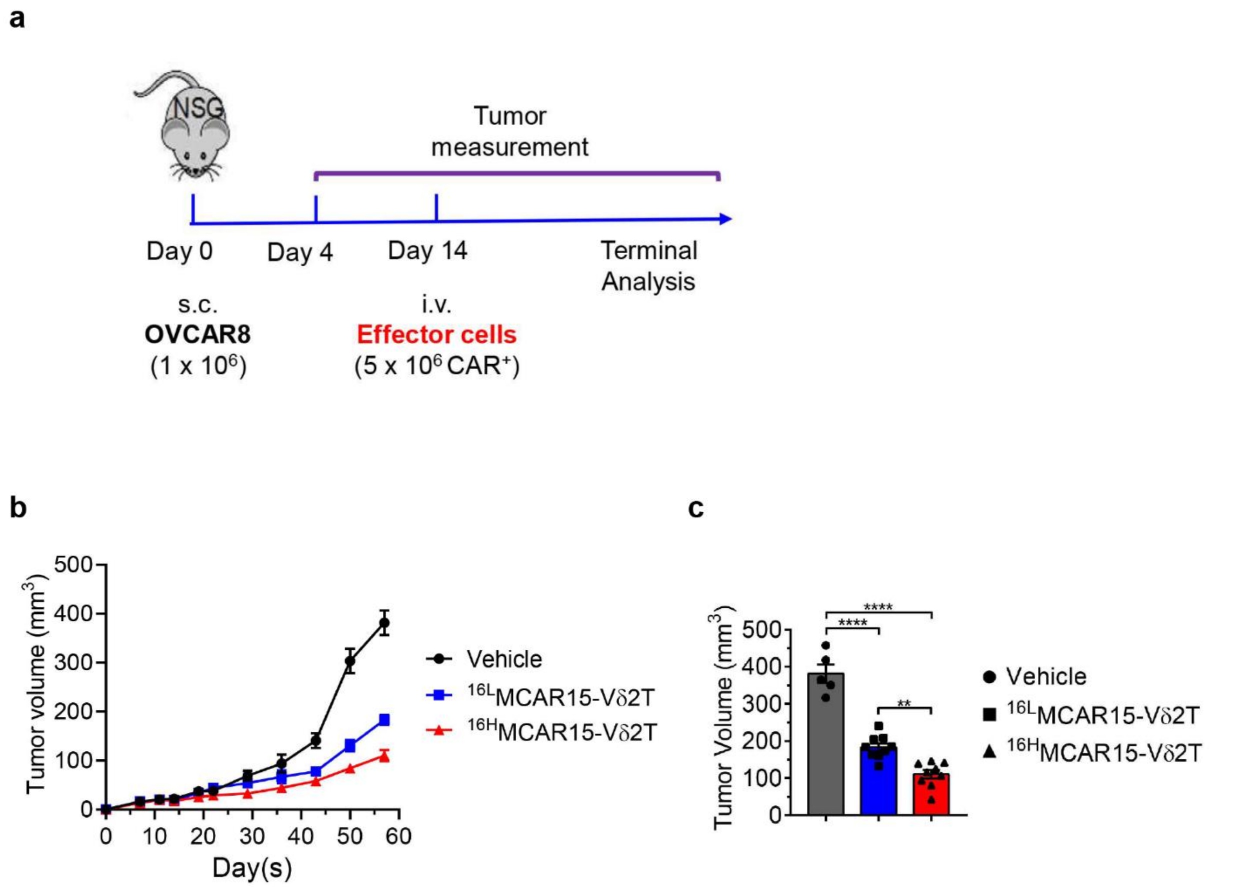

Supplementary Fig. 5 Comparing the *in vivo* antitumor efficacy of CD16^{Hi} and CD16^{Lo} MCAR15-Vδ2T cells. Vδ2 T cells were cultured from the CD16^{Hi} or CD16^{Lo} donor PBMCs and were engineered to express MCAR and IL-15. The resulting cell products are denoted as ^{16H}MCAR15-Vδ2T or ^{16H}MCAR15-Vδ2T cells, respectively. **a** Experimental design. Three experimental groups were included: Vehicle (mice receiving no effector cells; n = 5), ^{16L}MCAR15-Vδ2T (mice receiving ^{16H}MCAR15-Vδ2T cells; n = 9 from 3 different donors), and ^{16L}MCAR15-Vδ2T (mice receiving ^{16L}MCAR15-Vδ2T cells; n = 9 from 3 different donors). **b** Tumor growth over time. **c** Tumor sizes measured on day 57. Representative of 2 experiments. Data are presented as the mean ± SEM. **p < 0.01; ****p < 0.0001 by one-way ANOVA (**c**). Source data and exact p values are provided as a Source Data file.
