# Supplementary material for: Unlocking the potential of allogeneic Vδ2 T cells for ovarian cancer therapy through CD16 biomarker selection and CAR/IL-15 engineering
Source: Nat Commun. 2023 Nov 8;14:6942. doi: 10.1038/s41467-023-42619-2 (PMC10632431; doi:10.1038/s41467-023-42619-2)
Supplement: Supplementary file 3 — Reporting Summary [file 41467_2023_42619_MOESM3_ESM.pdf]

nature portfolio

Corresponding author(s): Lili Yang

Last updated by author(s): 9/13/2023

Reporting Summary

Nature Portfolio wishes to improve the reproducibility of the work that we publish. This form provides structure for consistency and transparency in reporting. For further information on Nature Portfolio policies, see our Editorial Policies and the Editorial Policy Checklist.

Statistics

For all statistical analyses, confirm that the following items are present in the figure legend, table legend, main text, or Methods section.

n/a	Confirmed	
☐	☒	The exact sample size (*n*) for each experimental group/condition, given as a discrete number and unit of measurement
☐	☒	A statement on whether measurements were taken from distinct samples or whether the same sample was measured repeatedly
☐	☒	The statistical test(s) used AND whether they are one- or two-sided<br>*Only common tests should be described solely by name; describe more complex techniques in the Methods section.*
☐	☒	A description of all covariates tested
☐	☒	A description of any assumptions or corrections, such as tests of normality and adjustment for multiple comparisons
☐	☒	A full description of the statistical parameters including central tendency (e.g. means) or other basic estimates (e.g. regression coefficient) AND variation (e.g. standard deviation) or associated estimates of uncertainty (e.g. confidence intervals)
☐	☒	For null hypothesis testing, the test statistic (e.g. *F*, *t*, *r*) with confidence intervals, effect sizes, degrees of freedom and *P* value noted<br>*Give P values as exact values whenever suitable.*
☒	☐	For Bayesian analysis, information on the choice of priors and Markov chain Monte Carlo settings
☒	☐	For hierarchical and complex designs, identification of the appropriate level for tests and full reporting of outcomes
☒	☐	Estimates of effect sizes (e.g. Cohen's *d*, Pearson's *r*), indicating how they were calculated

Our web collection on statistics for biologists contains articles on many of the points above.

Software and code

Policy information about availability of computer code

Data collection	Flow cytometry: MACSQuant Analyzer 10 Flow Cytometer (Miltenyi Biotec); FACSAria II Flow Cytometer (BD Biosciences); Bulk RNA-Seq: HiSeq3000 (Illumina); In vitro assays: Infinite M1000 microplate reader (Tecan); Bioluminescence live animal imaging: HTX imaging system (Spectral Instrument Imaging)
Data analysis	FlowJo Software V10 (BD Biosciences); GraphPad Prism 7 (GraphPad); Rstudio (Rstudio); AURA Imaging Software (Spectral Instrument Imaging); Microsoft Excel (Microsoft)

For manuscripts utilizing custom algorithms or software that are central to the research but not yet described in published literature, software must be made available to editors and reviewers. We strongly encourage code deposition in a community repository (e.g. GitHub). See the Nature Portfolio guidelines for submitting code & software for further information.

Data

Policy information about availability of data

All manuscripts must include a data availability statement. This statement should provide the following information, where applicable:

- Accession codes, unique identifiers, or web links for publicly available datasets
- A description of any restrictions on data availability
- For clinical datasets or third party data, please ensure that the statement adheres to our policy

The bulk RNAseq data sets generated in this study have been deposited in the GEO database under accession code GSE235755. The remaining data associated with this study are presented in the Article or Supplementary Information. Source data are provided with this paper. Further information and requests may be directed

to and will be fulfilled by the corresponding author, Lili Yang (liliyang@ucla.edu).

Research involving human participants, their data, or biological material

Policy information about studies with human participants or human data. See also policy information about sex, gender (identity/presentation), and sexual orientation and race, ethnicity and racism.

Reporting on sex and gender	N/A
Reporting on race, ethnicity, or other socially relevant groupings	N/A
Population characteristics	N/A
Recruitment	N/A
Ethics oversight	N/A

Note that full information on the approval of the study protocol must also be provided in the manuscript.

Field-specific reporting

Please select the one below that is the best fit for your research. If you are not sure, read the appropriate sections before making your selection.

[x] Life sciences [ ] Behavioural & social sciences [ ] Ecological, evolutionary & environmental sciences

For a reference copy of the document with all sections, see nature.com/documents/nr-reporting-summary-flat.pdf

Life sciences study design

All studies must disclose on these points even when the disclosure is negative.

Sample size	In our in vitro assays, we utilized three biological groups as cell donors, consisting of three donors with high CD16 expression and three donors with low CD16 expression, each with three technical replicates. This resulted in a total of nine replicates. During the study design phase, we aimed to include a minimum of three donors per group to ensure the robustness of our results. While having more than three donors would have been beneficial, it would have significantly increased the workload for each experiment. In our in vivo studies, we employed five mice per group to compare Vehicle, MCAR-T, MCAR-Vδ2T, and MCAR15-Vδ2T groups. Additionally, we used nine mice per group to compare CD16 high vs. CD16 low MCAR15-Vδ2T cells.
Data exclusions	No data were excluded for analysis.
Replication	All experiments were performed independently. Replicates of each individual experiment was stated in its figure legends. The experimental findings were reproduced with similar results.
Randomization	For in vivo studies, all mice received tumor cells at Day 0 then were randomized to split into different groups prior to therapeutic cells injection. All the in vitro and in vivo experiments each group was selected randomly.
Blinding	In our in vivo studies, group allocation was conducted in a blinded manner, with all investigators being unaware of the group identities. One individual was responsible for preparing therapeutic cells labeled as Group A, Group B, Group C, and Group D. Subsequently, another individual performed the injection of therapeutic cells without prior knowledge of the contents of each group. Finally, a third person conducted bioluminescence imaging (BLI) or tumor measurements.

Reporting for specific materials, systems and methods

We require information from authors about some types of materials, experimental systems and methods used in many studies. Here, indicate whether each material, system or method listed is relevant to your study. If you are not sure if a list item applies to your research, read the appropriate section before selecting a response.

Materials & experimental systems

n/a	Involved in the study
	☒ Antibodies
	☒ Eukaryotic cell lines
☒	☐ Palaeontology and archaeology
	☒ Animals and other organisms
☒	☐ Clinical data
☒	☐ Dual use research of concern
☒	☐ Plants

Methods

n/a	Involved in the study
☒	☐ ChIP-seq
	☒ Flow cytometry
☒	☐ MRI-based neuroimaging

Antibodies

Antibodies used

Antibodies for flow cytometry

Fluorochrome-conjugated antibodies specific for human APC/Cy7-CD45 (Cat. 304014, Clone H130, 1:100 dilution), PE/Cy7-TCRαβ (Cat. 306720, Clone IP26, 1:25 dilution), FITC-CD3 (Cat. 317306, Clone OKT3, 1:200 dilution), PB-CD3 (Cat. 317314, Clone OKT3, 1:100 dilution), FITC-CD27 (Cat. 356403, Clone M-T271, 1:100 dilution), APC/Cy7-CD45RA (Clone HI100, 1:200 dilution), FITC-CD56 (Cat. 318304, Clone HCD56, 1:10 dilution), APC-TCR Vγ9 (Cat. 331309, Clone B3, 1:100 dilution), PerCP-TCR Vδ2 (Cat. 331410, Clone B6, 1:100 dilution), APC-CD16 (Clone 3G8, 1:250 dilution), PE-CCR2 (Cat. 357206, Clone K036C2, 1:400 dilution), PE/Cy7-CCR4 (Cat. 359410, Clone L291H4, 1:500 dilution), FITC-CCR5 (Cat. 359120, Clone J418F1, 1:200 dilution), PE/Cy7-CXCR3 (Cat. 353720, Clone G025H7, 1:100 dilution), APC/Cy7-IFN-γ (Cat. 502529, Clone B27, 1:100 dilution), FITC-granzyme B (Cat. 372205, Clone QA16A02, 1:1000 dilution), PE/Cy7-perforin (Cat. 308125, Clone dG9, 1:50 dilution), PE-pSTAT5 (Tyr694, Cat. 936903, Clone A17016B, 1:50 dilution), PE/Cy7-Bcl-2 (Cat. 633511, Clone BCL/10C4, 1:100 dilution), APC-HER2 (Cat. 324407, Clone 24D2, 1:400 dilution), PB-CD14 (Cat. 301815, Clone 63D3, 1:1000 dilution), FITC-CD11b (Cat. 301330, Clone ICRF44, 1:10000 dilution), APC/Cy7-CD163 (Cat. 333622, Clone GHI/61, 1:500 dilution), APC-CD206 (Cat. 321110, Clone 15-2, 1:500 dilution), and APC-Streptavidin (Cat. 405207, 1:1000 dilution) to bind Biotinylated Human Mesothelin for MCAR staining were all purchased from BioLegend.

Biotinylated Human Mesothelin (Cat. MSN-H82E9, 1:400 dilution) was purchased from ACROBiosystems. Fluorochrome-conjugated antibody specific for human APC-Mesothelin (Cat. FAB32652A, Clone 420411, 1:100 dilution) was purchased from R&D Systems. Fluorochrome-conjugated antibody specific for human FITC-Bcl-xL (Cat. MA5-28637, Clone 7B2.5, 1:100 dilution) was purchased from Thermo Fisher Scientific. Fluorochrome-conjugated antibody specific for human FITC-TCRγ/δ (Cat. 347903, Clone 11F2, 1:15 dilution) was purchased from BD Biosciences. Fluorochrome-conjugated antibody specific for human PE-TCR Vδ1 (Cat. 130-120-580, Clone REA173, 1:500 dilution) was purchased from Miltenyi Biotec. Fixable Viability Dye eFluor506 (e506, Cat. 65-0866-18) was purchased from Affymetrix eBioscience. Mouse Fc Block (anti-mouse CD16/32, Cat. 553142, Clone 2.4G2, 1:50 dilution) was purchased from BD Biosciences, and human Fc Receptor Blocking Solution (TrueStain FcX, Cat. 422302, 1:25 dilution) was purchased from BioLegend. InVivoSIM anti-human HER2 (Trastuzumab Biosimilar, Cat. SIM0005) was purchased from BioXCell.

Antibodies for ELISA

The capture (Cat. 551221, Clone NIB42, 1:250 dilution) and biotinylated (Cat. 554550, Clone 4S.B3, 1:500 dilution) pairs for detecting IFN-γ were purchased from BD Biosciences. The HRP-Avidin conjugate (Cat. 405103, 1:1000 dilution) and the human IFN-γ ELISA standards (Cat. 570209) were purchased from BioLegend.

Antibodies for PBMC-T cell culture

Anti-CD3 monoclonal antibody OKT3 (BioLegend; Cat. 317325).

Validation

All the antibodies used are from commercial sources and have been validated by the vendors. Validation data are available on the manufacturer's website. Each antibody purchased for this study has relevant citation and profile.

Eukaryotic cell lines

Policy information about cell lines and Sex and Gender in Research

Cell line source(s)	Human embryonic kidney 293T/17 (HEK-293T/17, ATCC; Cat. CRL-11268), and human ovarian cancer cell lines OVCAR3 (ATCC; Cat. HTB-161), OVCAR8 (NIH; Cat. CVCL_1629), and SKOV3 (ATCC; Cat. HTB-77) were all purchased from American Type Culture Collection (ATCC) or obtained from National Institutes of Health (NIH) under MTA.<br><br>To make stable tumor cell lines overexpressing a firefly luciferase and enhanced green fluorescence protein (FG) dual reporter, the parental tumor cell lines were transduced with a Lenti/FG vector encoding the FG dual reporter. 72 h-post lentivector transduction, cells were subjected to flow cytometry sorting to isolate gene-engineered cells for making stable cell lines. Three FG labeled stable tumor cell lines were generated for this study, including OVCAR3-FG, OVCAR8-FG, and SKOV3-FG.<br><br>One additional cell line that overexpresses the FG dual reporter as well as a knockout of mesothelin (MSLN) tumor antigen, KO OVCAR3-FG, was generated for this study. Briefly, OVCAR3-FG cells were electroporated with a CRISPR-Cas9/MSLN-sgRNA complex composed of pre-mixed Cas9-NLS protein (4 µL at 6.5 µg/µL; UC Berkeley) and MSLN-sgRNA (1 µL at 100 µM; sequence: GGAAGCCAAGGAGUUGGCGA; Synthego). For electroporation, tumor cells were pulsed twice at 1170 V for 30 ms in a Neon Transfection System (Thermo Fisher Scientific; Cat. MPK5000) following manufacturer's protocol. 72 h post electroporation, the engineered OVCAR3-FG cells were subjected to flow cytometry sorting to isolate MSLN-KO OVCAR3-FG (KO OVCAR3-FG) cell line.
Authentication	HEK-293T/17 cells were authenticated by ATCC.<br>OVCAR3 cells were authenticated by ATCC.<br>OVCAR8 cells were authenticated by NIH.<br>SKOV3 cells were authenticated by ATCC.<br>All the FG engineered cells and MSLN knockout OVCAR3-FG cells were not authenticated.
Mycoplasma contamination	All cell lines were tested negative for mycoplasma contamination.
Commonly misidentified lines<br>(See ICLAC register)	No misidentified lines were used.

Animals and other research organisms

Policy information about studies involving animals; ARRIVE guidelines recommended for reporting animal research, and Sex and Gender in Research

Laboratory animals	NOD.Cg-PrkdcSCIDIl2rgtm1Wjl/SzJ (NOD/SCID/IL-2Rγ-/-, NSG) mice were maintained in the animal facilities of the University of California, Los Angeles (UCLA) under the following housing conditions: temperature ranging from 68°F to 79°F, humidity maintained at 30% to 70%, a light cycle of On at 6:00 am and Off at 6:00 pm, and room pressure set to negative. 6-10 weeks old female mice were used for all experiments unless otherwise indicated.
Wild animals	No wild animals were used in this study.
Reporting on sex	Female mice were used in this study.
Field-collected samples	No field-collected samples were used in this study.
Ethics oversight	All mice were approved by the Institutional Animal Care and Use Committee (IACUC) of UCLA, and all experiments were conducted in accordance with the animal care and use regulations of the Division of Laboratory Animal Medicine (DLAM) at the UCLA.

Note that full information on the approval of the study protocol must also be provided in the manuscript.

Flow Cytometry

Plots

Confirm that:

- [x] The axis labels state the marker and fluorochrome used (e.g. CD4-FITC).
- [x] The axis scales are clearly visible. Include numbers along axes only for bottom left plot of group (a 'group' is an analysis of identical markers).
- [x] All plots are contour plots with outliers or pseudocolor plots.
- [x] A numerical value for number of cells or percentage (with statistics) is provided.

Methodology

Sample preparation	Flow cytometry surface stainings and intracellular stainings were performed following standard protocols, as well as specific instructions provided by a manufacturer for particular antibodies.

Instrument	MACSQuant Analyzer 10 Flow Cytometer (Miltenyi Biotec); FACSAria II Flow Cytometer (BD Biosciences).
Software	FlowJo software V10 (BD Biosciences).
Cell population abundance	50000 to 200000 cells per sample were used for staining procedure.
Gating strategy	SSC-A/FSC-A, SSC-W/SSC-H and FSC-W/FSC-H were used to identify singlets. Fixable Viability Dye eFluor 506 (Biolegend) was used to exclude dead cells in flow analysis. Gating strategy for each cell subtype in each experiment was described in the article.

[x] Tick this box to confirm that a figure exemplifying the gating strategy is provided in the Supplementary Information.
